# Histone Deacetylase Inhibitors Trichostatin A and MCP30 Relieve Benzene-Induced Hematotoxicity via Restoring Topoisomerase IIα

**DOI:** 10.1371/journal.pone.0153330

**Published:** 2016-04-08

**Authors:** Jingjing Chen, Zhouyi Zheng, Yi Chen, Jiaqi Li, Shanhu Qian, Yifen Shi, Lan Sun, Yixiang Han, Shenghui Zhang, Kang Yu

**Affiliations:** 1 Department of Hematology, the First Affiliated Hospital of Wenzhou Medical University, Wenzhou, 325015, China; 2 Laboratory of Internal Medicine, the First Affiliated Hospital of Wenzhou Medical University, Wenzhou, 325015, China; University of Macau, MACAO

## Abstract

Dysfunction of histone acetylation inhibits topoisomerase IIα (Topo IIα), which is implicated in benzene-induced hematotoxicity in patients with chronic benzene exposure. Whether histone deacetylase (HDAC) inhibitors can relieve benzene-induced hematotoxicity remains unclear. Here we showed that hydroquinone, a main metabolite of benzene, increased the HDAC activity, decreased the Topo IIα expression and induced apoptosis in human bone marrow mononuclear cells *in vitro*, and treatment with two HDAC inhibitors, namely trichostatin A (TSA) or a mixture of ribosome-inactivating proteins MCP30, almost completely reversed these effects. We further established a benzene poisoning murine model by inhaling benzene vapor in a container and found that benzene poisoning decreased the expression and activity of Topo IIα, and impaired acetylation of histone H4 and H3. The analysis of regulatory factors of Topo IIα promoter found that benzene poisoning decreased the mRNA levels of SP1 and C-MYB, and increased the mRNA level of SP3. Both TSA and MCP30 significantly enhanced the acetylation of histone H3 and H4 in Topo IIα promoter and increased the expression and activity of Topo IIα in benzene poisoning mice, which contributed to relieve the symptoms of hematotoxicity. Thus, treatment with HDAC inhibitors represents an attractive approach to reduce benzene-induced hematotoxicity.

## Introduction

Benzene, a ubiquitous environmental pollutant due to its wide range of practical applications as an industrial solvent or as a starting material in making other chemicals worldwide, is an established human hematotoxicant and leukemogen. Chronic exposure to benzene commonly causes bone marrow suppression that often initially manifests clinically decreased peripheral blood cell counts, and ultimately leads to the onset of various disorders, including pancytopenia, aplastic anemia, and myelogenous leukemia [1–3]. Hematopoietic stem and progenitor cells are sensitive targets for cytotoxicity and genotoxicity induced by benzene [4]. Benzene is metabolized in the liver and bone marrow, and its metabolites can damage hematopoietic cells through multiple mechanisms, including apoptosis, gene-expression alteration, and epigenetic regulation [5,6].

DNA topoisomerases are conserved nuclear enzymes that modify DNA topology in a very precise fashion. Therein, topoisomerase IIα (Topo IIα) has been shown as a key player in several crucial cell processes, including replication, transcription, chromosome separation and segregation [7]. Accumulating evidences have demonstrated that active metabolites of benzene including hydroquinone (HQ) inhibit Topo IIα and subsequently lead to DNA damage, cell apoptosis or aberration, and eventually carcinogenesis [8–11]. Our previous study has also demonstrated that the expression and activity of Topo IIα are reduced in patients with chronic benzene exposure [12], supporting the notion that benzene can induce hematotoxicity through inhibition of Topo IIα.

The acetylation of histones is the first epigenetic modification connected with biological activity [13]. Histone acetylation often up-regulates the transcriptional activity of the gene while histone deacetylation always silences the gene [14,15]. Since histone has been shown to be one of the potential targets of benzene metabolites [16], it can be speculated that exposure to benzene causes the onset of histone modifications in hematopoietic cells. Our previous study has found that alterations in histone acetylation and methylation of Topo IIα promoter may result in reduced expression and activity of Topo IIα [12]. Histone deacetylases (HDACs) as a class of enzymes affect the acetylation status of histones and other vital cellular proteins, and their inhibitors such as trichostatin (TSA) and etacrox result in a dramatic increase of histone acetylation [17,18]. Our previous study has shown that MCP30, a mixture contains two ribosome-inactivating proteins alpha-momorcharin and beta-momorcharin isolated from bitter melon seeds, significantly inhibits the activity of HDAC-1 and promotes the acetylation of histone H3 and H4 [19]. Whether MCP30 affects the expression and activity of Topo IIα remains unclear.

In addition to epigenetic modification, other regulatory factors including SP1 [20], ATF-2 [21], SP3 [22], C-MYB [23], and ICBP90 [24] also affect Topo IIαpromoter activity. Elevated mRNA levels of SP3 and p53, and decreased mRNA levels of SP1, NF-Y, NF-M, C-MYB, and C-JUN, have been shown in patients with chronic benzene exposure compared with healthy volunteers, which may also lead to reduced expression and activity of Topo IIα [12].

In the present study, we demonstrated that HQ increased the HDAC activity, decreased the expression of Topo IIα, and induced apoptosis in human bone marrow mononuclear cells, and TSA or MCP30 treatment almost completely reversed these effects. Long-term inhalation of benzene in mice resulted in decreased expression and activity of Topo IIα through reduced acetylation of its promoter and alterations of other regulatory factors, and which contributed to the suppression of bone marrow hematopoietic function, and TSA or MCP30 treatment reduced benzene-induced hematotoxicity.

## Materials and Methods

### Preparation of human bone marrow mononuclear cell suspension

Bone marrow aspirates were obtained from five healthy donors, and further separated for mononuclear cells using Ficoll-Hypaque. The mononuclear cells were suspended at 1 × 10^6^/ml in RPMI 1640 medium supplemented with 10% fetal bovine serum. The cell viabilities were assessed using trypan blue dye exclusion staining and only viability more than 95% were used for further experiments. This study was carried out with approval from the Ethics Committee of the first affiliated hospital of Wenzhou medical university and written consents were obtained from all subjects participated in this study in accordance with the Declaration of Helsinki protocol.

### HDAC activity assay

The activity of total HDACs was determined using HDACs colorimetric assay kit (Millipore, Bedford, MA, USA) according to the manufacturer’s protocol. Briefly, 20 μl of sample was added into each well with 10 μl of 2 × HDAC Assay Buffer and 10 μl of HDAC substrate containing 4 mM TSA. The reaction was carried out at 37°C for 1 h and subsequently terminated with 10 μl of diluted activator solution. The absorbance (OD) was read at 405 nm using an ELISA reader (Elx800; Bio-Tek, Winooskie, VT, USA). HDAC activity = (OD_405 nm, sample_—OD_405 nm, control_) / protein content (μg).

### Flow cytometric analysis

Annexin V-FITC/PI double staining was used to evaluate the apoptosis according to our previously described method [25,26]. Briefly, human bone marrow mononuclear cells were treated with or without 100 μM HQ for 24 h, in the presence or absence of TSA (0.5 μM) or MCP30 (1 μg/ml). Then, the cells were harvested and washed and incubated in cold buffer and stained for 10 min with FITC-labeled Annexin V and PI (MultiSciences Biotech, Hangzhou, China), followed by analysis on a flow cytometry (FACSCalibur; BD, Mountain View, CA, USA). Additionally, human bone marrow mononuclear cells were treated with or without 200 nM daunorubicin for 48 h, and subsequently stained with FITC-labeled Annexin V and analyzed for apoptosis on a flow cytometry.

### Animals and grouping

All experimental procedures were approved by the Institutional Animal Committee of Wenzhou Medical University. All mice received care throughout the experiment in accordance with “Guide for the Care and Use of Laboratory Animals”. Six- to 7-week-old male CD1 mice were purchased from Vital River Laboratory Animal Technology Co. Ltd. (Beijing, China) and housed in specific pathogen-free facilities and maintained under controlled temperatures (20–22°C), humidity (45–55%) and light (12 h light/dark cycle) conditions with standard mouse chow and water. According to weights of the mice, they were divided into 6 groups randomly, then subsequently inhaled benzene vapor or not, with or without administration of TSA or MCP30. Mice were placed in a container to inhale 300 parts per million (ppm) benzene vapor for 6 h per day, 5 days per week for 8 weeks according to the methods previously described [27]. Meanwhile, TSA or MCP30 was given by intraperitoneal injection at a dose of 1 mg/kg prior to each inhalation of benzene. Before benzene inhalation and 2 days after completion, blood samples of each mouse were obtained from caudal or orbital veins. No mice died during the experiment, and ultimately, all mice were killed by cervical dislocation and their bilateral femurs and tibias were separated. Bone marrow smears were obtained by flushing femurs and tibias with PBS. The histopathological studies were carried out according to our previously reported method [28].

### Extraction of nuclear proteins

The nuclear proteins were extracted from bone marrow mononuclear cells (5–10 × 10^6^) using NE-PER Nuclear and Cytoplasmic Extraction Kit (Thermo Fisher Scientific, Rockford, IL, USA) according to the manufacturer's instruction. The concentration of nuclear protein was determined using a BCA assay (Beyotime, Haimen, Jiangsu,China).

### Western blot analysis

The nuclear protein was extracted from bone marrow mononuclear cells and subsequently denatured and separated on 7.5% sodium dodecyl sulfate polyacrylamide gel electrophoresis (SDS-PAGE) and blotted onto a polyvinylidene difluoride (PVDF) membrane (Millipore).The membrane was blocked using 5% nonfat dry milk for 1 h at room temperature, and incubated with antibodies against Topo IIα, histone H3 or TBP (Cell Signaling Technologies, Beverly, MA, USA) at 4°C overnight. After washing, the membrane was subsequently incubated with HRP-labeled goat anti-rabbit antibody at room temperature for 1 h. After washing, membranes were developed using enhanced chemiluminescence and the optical densities of the bands were determined using Image J software (NIH, Bethesda, MD, USA).

### Topo IIα activity assay

The activity of Topo IIα was determined using a Topo II Assay Kit (TopoGen, Colombus, OH, USA) according to the principle which Topo IIα converts catenated kinetoplast DNA (kDNA) into the form of nicked open circular minicircles and fully closed circular rings. The resulting products were visualized on a 1% agarose gel following 0.5% ethidium bromide staining. The kDNA is a large network of high molecular weight DNA that does not penetrate an agarose gel, thus, it could be used as its own blank control.

### Reverse transcriptase-polymerase chain reaction (RT-PCR)

Total RNA was extracted from mouse bone marrow mononuclear cells using TRIzol reagent (Invitrogen, Carlsbad, CA, USA) and reverse transcribed into cDNA using the ReverTra Ace qPCR RT kit (Toyobo, Osaka, Japan), and cDNA was amplified using PCR Master Mix (Tiangen Biotech, Beijing, China) with specific primer pairs. The primer sequences and PCR product sizes were detailed in Table 1. All reactions were performed in triplicates and normalized using GAPDH as an endogenous control gene. PCR products were separated on 1.5% agarose gel and analyzed using the Quantity One Gel Analysis System. Relative level of gene expression was defined as a ratio of the target gene to GAPDH gene expression.

**Table 1:** The sequences of the primers used for PCR and the corresponding product sizes.

genes	primers(5’ - 3’)	products (bp)
SP1	forward: AAGGATGCGGCAAAGTAT	123
	reverse: CGTCCGAACGTGTAAAGC	
ATF-2	forward: GGAAAGTGTGGGTTCAGTCC	150
	reverse: GGAAAGTGTGGGTTCAGTCC	
SP3	forward: GTGCTCGCATCTGTGGAA	138
	reverse: GTCTTGATTGCTGGTGGC	
C-MYB	forward: GCAGGCATTACCAACACAGA	114
	reverse: TCTCCCAAACAGGAAACAGG	
ICBP90	forward: TCCAGTGCCGTTAAGACCTC	102
	reverse: ACGGACATTCTTGGCTTTGA	
Topo IIα	forward: GAGGCTTCCAGCAGATTAGC	107
	reverse: CACCACATCAACGAGTTTGC	
GAPDH	forward: GGGCATCTTGGGCTACACT	209
	reverse: GGTCCAGGGTTTCTTACTCC	

### Chromatin immunoprecipitation (ChIP) assay

ChIP assay was carried out using a commercial kit (Millipore) according to our previously described method [12]. Briefly, chromatin was fragmented and subsequently immunoprecipitated with antibodies against acetyl-histone H4 (06–866; Millipore) or acetyl-histone H3 (06–599; Millipore). Then, the immunoprecipitated DNAs were determined for Topo IIα promoter by PCR. The forward primer was 5’-GCTCTTGTAGATTCCCTCAGC-3’ and the reverse primer was 5’-AGCAGCACTAACACTCCCTGT-3’, with an expected PCR product of 130 bp. PCR products were separated on 1.5% agarose gels and analyzed using the Quantity One Gel Analysis System.

### Peripheral blood cell counts

Blood cell counts were determined based on a previous reported method [28,29]. Briefly, the hemoglobin concentration was measured using a hemoglobin analyzer (Mission, Sichuan, China). White blood cells were quantified under a light microscopy after blood was diluted in 3% acetic acid. Platelets were quantified under a phase-contrast microscope after blood was diluted with 1% ammonium oxalate.

### Statistical analysis

Data were expressed as mean ± SEM and analyzed by two-way ANOVA using SPSS version 19.0 (SPSS Inc., Chicago, IL, USA). Differences were considered statistically significant at *P* < 0.05.

## Results

### TSA or MCP30 treatment decreases the activity of HDAC, increases the expression of Topo IIα and reduces apoptosis in human bone marrow mononuclear cells induced by HQ

**Fig 1:**
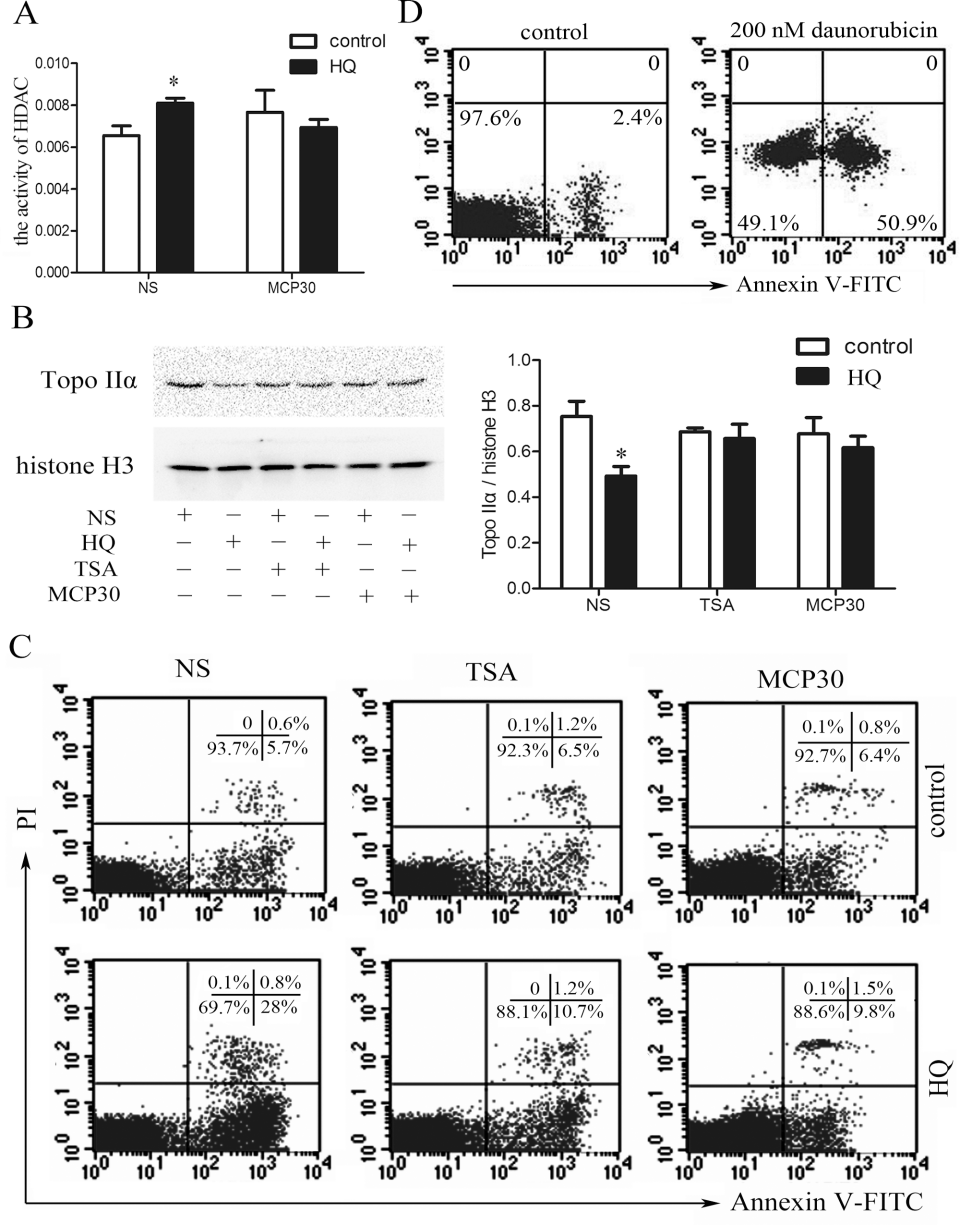
TSA or MCP30 restores the HQ-induced increased HDAC activity, decreased Topo IIα expression, and the resulting apoptosis in human bone marrow mononuclear cells. (**A—C**) Mononuclear cells were isolated from bone marrow aspirates from four healthy donors and subsequently treated with or without 100 μM HQ, in the presence or absence of TSA (0.5 μM) or MCP30 (1 μg/ml). After 24 h, the activity of HDAC (**A**), the expression of Topo IIα (**B**), and apoptosis (**C**) were determined using HDAC activity assay kit, western blot, and Annexin V/PI double staining, respectively. NS stands for normal saline. Histone H3 was used as a loading control for nuclear protein in western blot analysis. (D) Human bone marrow mononuclear cells were treated with or without a Topo IIα inhibitor daunorubicin (200 nM) for 48 h and apoptosis was measured using Annexin V staining. Statistical data and representatives of four independent experiments from different healthy donors were shown. *P < 0.05, compared to the respective control group.

HQ, a main metabolite of benzene in humans, increased the activity of HDAC in human bone marrow mononuclear cells, and MCP30, a mixture of MAP30 and α-MMC in half, attenuated the increased activity of HDAC induced by HQ (Fig 1A). Since our previous study has shown a decreased expression and activity of Topo IIα in patients with chronic benzene exposure [12], we further evaluated the effect of HQ on the expression of Topo IIα in human bone marrow mononuclear cells *in vitro*. As shown in Fig 1B, HQ decreased the expression of Topo IIα, and two HDAC inhibitors, TSA or MCP30, almost completely reversed this effect. Moreover, HQ also induced a significant increase in apoptosis in bone marrow mononuclear cells with almost no effect on the cells after treatment with TSA or MCP30 (Fig 1C). Obviously, treatment with a Topo IIα inhibitor daunorubicin [30] induced apoptosis in human bone marrow mononuclear cells (Fig 1D). Taken together, the data suggest that HQ increases the activity of HDAC, decreases the expression of Topo IIα, and induces apoptosis in human bone marrow mononuclear cells, and HDAC inhibitors treatment can reverse these effects.

### TSA or MCP30 restores decreased Topo IIα expression induced by benzene

**Fig 2:**
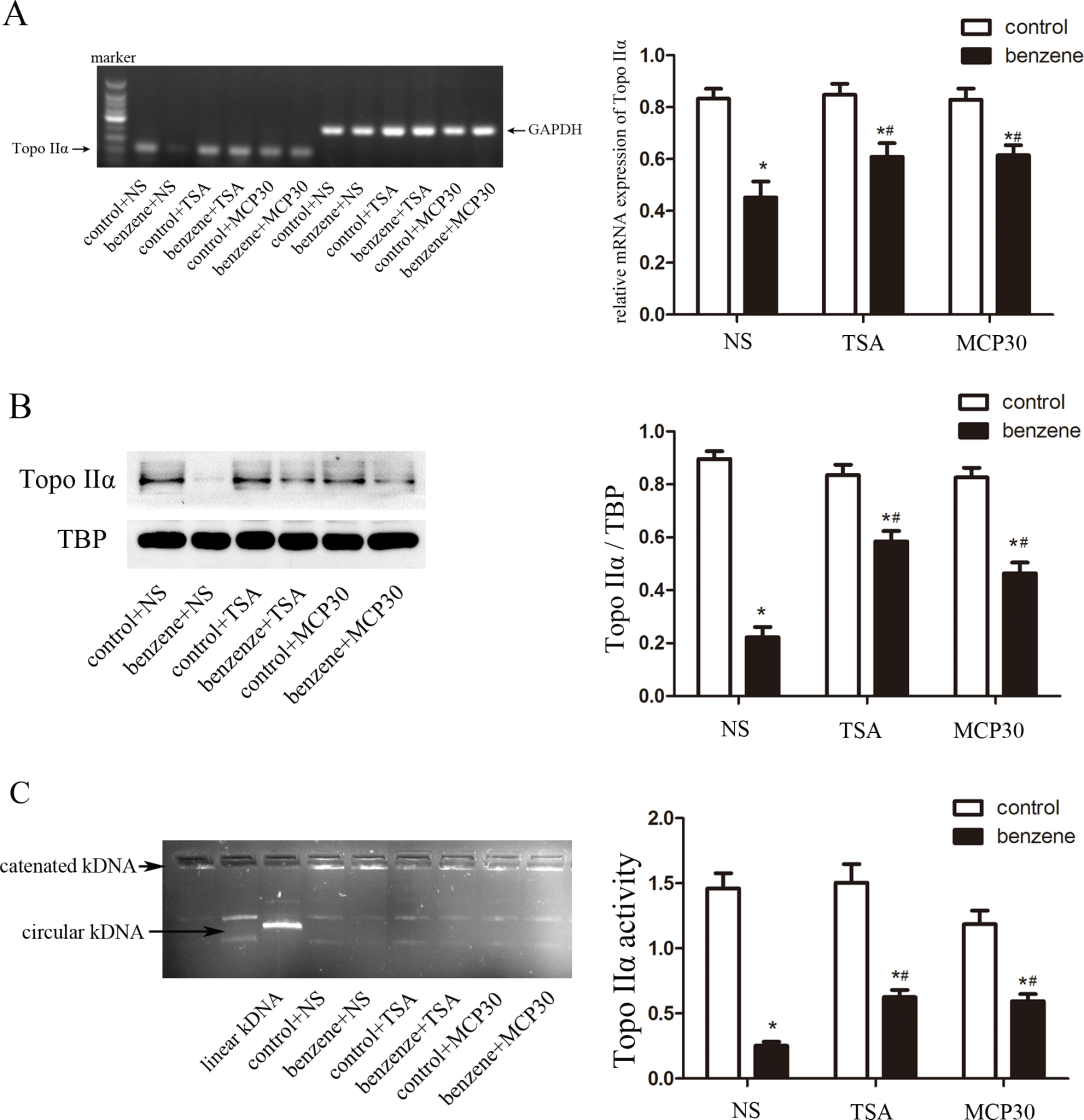
TSA or MCP30 restores the expression and activity of Topo IIα in benzene poisoning mice. Mice inhaled 300 ppm benzene vapor for 8 weeks and TSA or MCP30 was intraperitoneally injected at a dose of 1 mg/kg. After all mice were killed, bone marrow mononuclear cells were separated and measured the expression of Topo IIα including mRNA (**A**), protein (**B**), and activity (**C**) using RT-PCR, western blot, and Topo II activity assay kit, respectively. GAPDH was used as a reference gene in RT-PCR analysis, and TBP was used as loading control in western blot analysis. Images representing 8 mice per group were shown in left column and statistical data were shown in right column. *P < 0.05, compared to the control group; ^#^*P* < 0.05, compared to the benzene alone-treated group.

Benzene active metabolites including HQ have been shown to inhibit the expression and activity of Topo IIα *in vitro* [8]. However, whether Topo IIα is implicated in benzene-induced hematotoxicity is not well clarified. As shown in Fig 2A, inhalation of benzene significantly decreased the mRNA level of Topo IIα in bone marrow mononuclear cells from benzene poisoning mice compared with those from the control mice. Similar results were also observed in the protein level and enzyme activity of Topo IIα (Fig 2B and 2C). Taken together, the expression and activity of Topo IIα were prominently decreased in bone marrow mononuclear cells from benzene poisoning murine model.

HDAC inhibitors alone did not affect the mRNA and protein levels, and enzyme activity of Topo IIα (Fig 2A–2C). However, HDAC inhibitors TSA or MCP30 restored the decreased expression and activity of Topo IIα of bone marrow mononuclear cells in benzene poisoning murine model (Fig 2A–2C). Conclusively, these data suggest that HDAC inhibitors restore the benzene-induced decreased expression and activity of Topo IIα *in vivo*.

### TSA or MCP30 restores benzene-induced decreased acetylation of Topo IIα promoter

**Fig 3:**
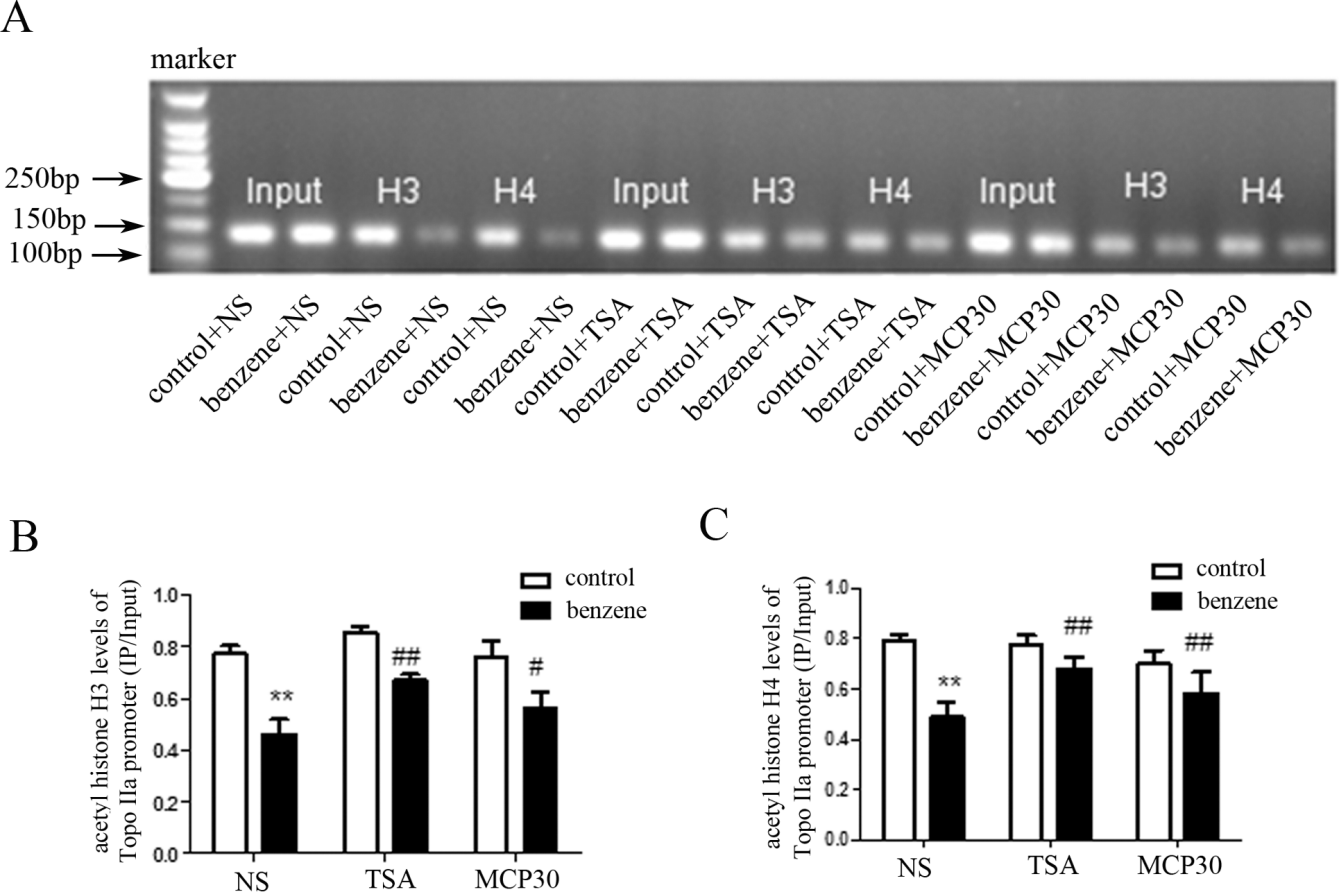
TSA or MCP30 increases the decreased acetylation of Topo IIα promoter in benzene poisoning mice. After all mice were killed, bone marrow mononuclear cells were separated and histone acetylation of Topo IIα promoter was assessed with chromatin immunoprecipitation (ChIP) assay using anti-acetylated histone H3 and anti-acetylated histone H4. (**A**) Representatives were shown for acetylation levels of histone H3 and histone H4 in the Topo IIα promoter. (**B**) Statistical data showed the acetylation levels of histone H3 in the Topo IIα promoter were shown. (**C**) Statistical data showed the acetylation levels of histone H4 in the Topo IIα promoter. ^**^P < 0.01, compared to the control group; ^#^*P* < 0.05, ^##^*P* < 0.01, compared to the benzene alone-treated group.

In order to investigate the mechanisms by which the expression of Topo IIα was reduced in bone marrow mononuclear cells from benzene poisoning murine model, the acetylation of Topo IIα promoter was assessed. Using ChIP with anti-acetyl-histone H3 and anti-acetyl-histone H4, we found that acetyl-histone H3 and acetyl-histone H4 bound to Topo IIα promoter were significantly decreased in bone marrow mononuclear cells from benzene alone-treated mice compared to those from control mice (Fig 3). TSA or MCP30 alone did not influence the acetylation of Topo IIα promoter (Fig 3). Compared with benzene alone-treated mice, acetyl-histone H3 and acetyl-histone H4 bound to Topo IIα promoter were markedly increased in bone marrow mononuclear cells from benzene and TSA-treated mice as well as benzene and MCP30-treated mice (Fig 3). Taken together, these data suggest that TSA or MCP30 can restore the decreased acetylation of Topo IIα promoter induced by benzene exposure.

### TSA or MCP30 affects the mRNA levels of regulatory factors of Topo IIα promoter

**Fig 4:**
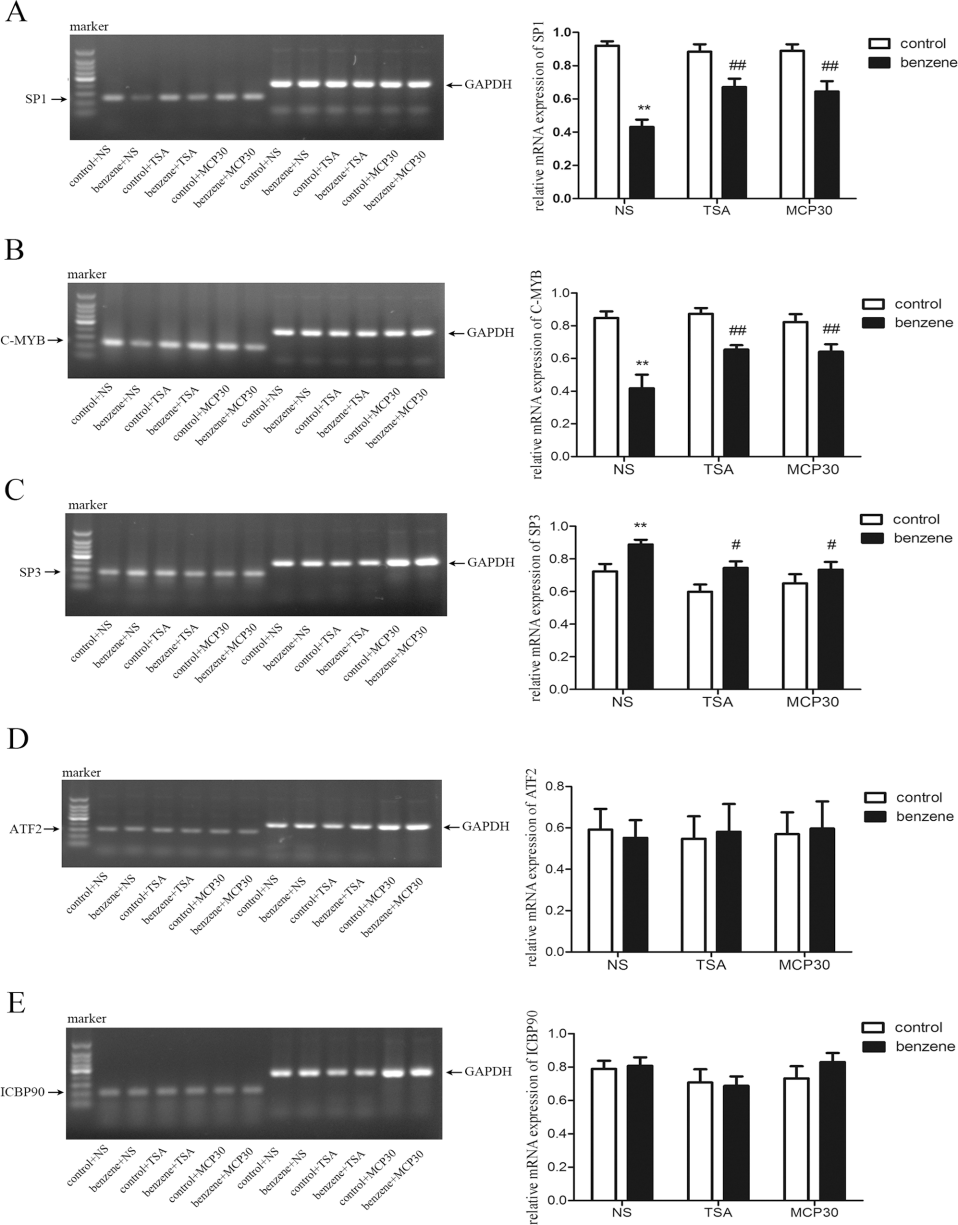
TSA or MCP30 alters the mRNA levels of regulatory factors in the Topo IIα promoter in benzene poisoning mice. After all mice were killed, bone marrow mononuclear cells were separated and the mRNA levels of regulatory factors, including SP1 (**A**), C-MYB (**B**), SP3 (**C**), ATF-2 (**D**), and ICBP90 (**E**) were determined using RT-PCR. Relative mRNA levels were defined as a ratio of the targeted gene to GAPDH. Representatives of 6 groups with 8 mice each group were shown in left column and statistical data were shown in right column. ^**^P < 0.01, compared to the control group. ^#^*P* < 0.05, ^##^*P* < 0.01, compared to the benzene alone-treated group.

To explore potential involvement of other Topo IIα promoter regulatory factors besides acetylation of Topo IIα promoter, the mRNA levels of SP1, ATF-2, SP3, C-MYB and ICBP90 were examined in bone marrow mononuclear cells from all mice. As shown in Fig 4A and 4B, benzene alone treatment resulted in a significant reduction in the mRNA expression of SP1 and C-MYB compared to the control mice. Compared with benzene alone-treated mice, TSA or MCP30 treatment increased the mRNA levels of SP1 and C-MYB (Fig 4A and 4B). Meanwhile, the mRNA level of SP3 was increased in benzene alone-treated mice compared to the control mice, and both TSA and MCP30 decreased the up-regulated mRNA level of SP3 induced by benzene (Fig 4C). Treatment with benzene, TSA or MCP30 did not influence the mRNA levels of ATF-2 and ICBP90 in all mice (Fig 4D and 4E). Taken together, these data suggest that treatment with TSA or MCP30 also results in alterations of regulatory factors of Topo IIα promoter induced by benzene.

### TSA or MCP30 relieves benzene-induced hematotoxicity

**Fig 5:**
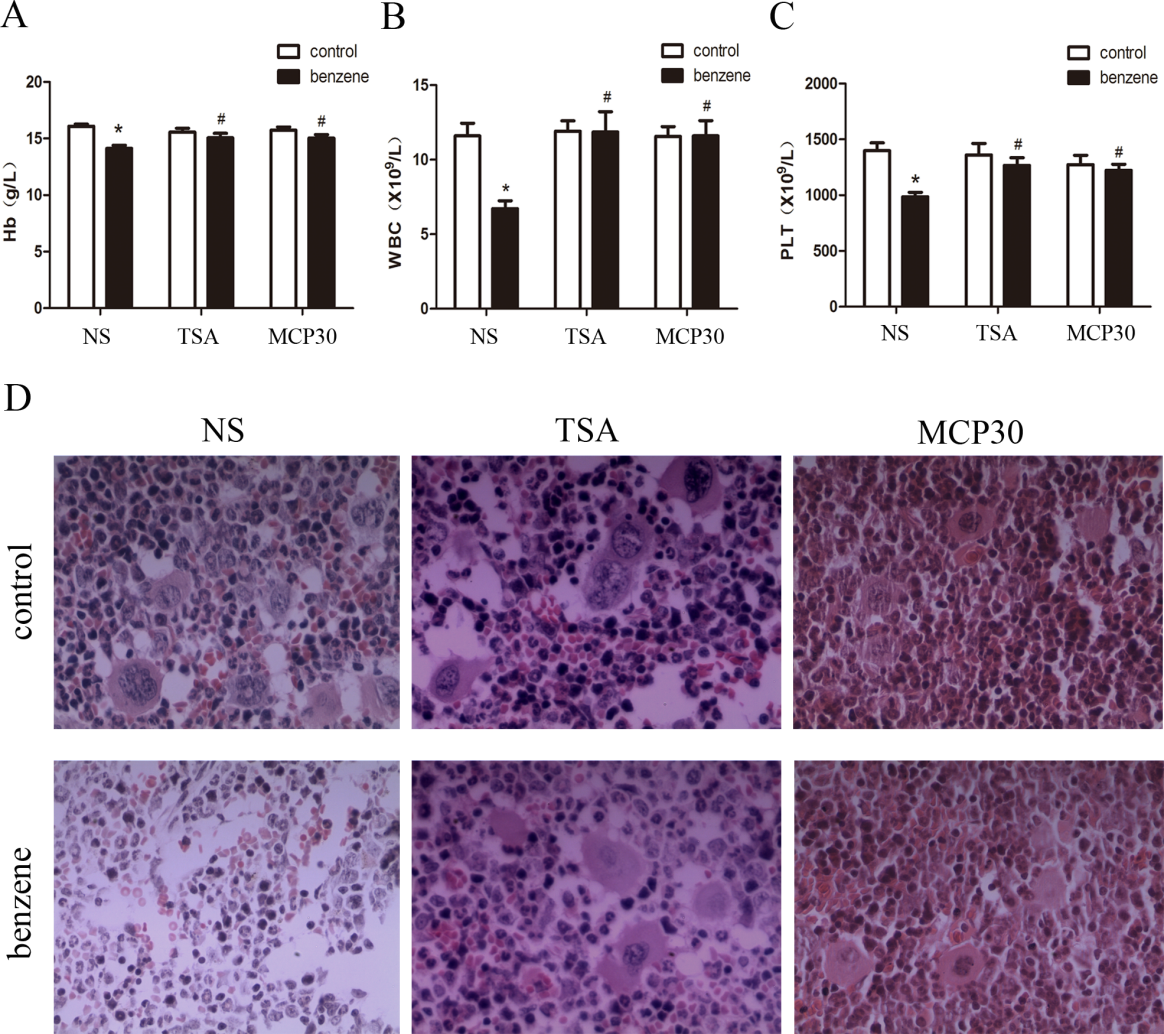
TSA or MCP30 relieves the benzene-induced hematotoxicity in benzene poisoning mice. After the completion of benzene inhalation, peripheral blood was obtained from each mouse by tail vein bleeding and the mice were subsequently killed. (**A—C**) Benzene alone treatment lowered the level of hemoglobin (Hb), and the number of white blood cells (WBC) and platelet (PLT) counts, and TSA or MCP30 reversed the above indicators in benzene-treated mice. Statistical data of 6 groups with 8 mice each group was shown. ^**^P < 0.01, compared to the control group; ^#^*P* < 0.05, ^##^*P* < 0.01, compared to the benzene alone-treated group. (**D**) The femurs were separated and embedded in paraffin and subsequently cut into slices with a thickness of 5 μm. After hematoxylin and eosin staining, femoral morphology was observed under a light microscope at a magnification of 400. Images shown were representative of at least three mice each group.

As duration of benzene inhalation increased, only benzene alone-treated mice gradually displayed the symptoms including depression, loss of appetite, dull sparse fur, and weight loss. Peripheral blood cell counts and pathological morphology are two well-known indicators to reflect the hematopoietic function of bone marrow. As shown in Fig 5A–5C, benzene alone resulted in a significant decrease in peripheral blood cell counts, including the level of hemoglobin, the numbers of white blood cells and platelets, compared to the control mice. Pathological morphology analysis of bone marrow had also demonstrated that there were a decrease of hematopoietic cells and an increase of non-hematopoietic cells in benzene alone-treated mice compared to the control mice (Fig 5D). Intraperitoneal injection of TSA or MCP30 alone did not affect peripheral blood cell counts and pathological morphology of bone marrow (Fig 5). TSA or MCP30 treatment led to an almost complete recovery of peripheral blood cell counts in mice induced by benzene (Fig 5A–5C). Taken together, these data suggest that TSA or MCP30 treatment can relieve benzene-induced hematotoxicity.

## Discussion

The effect of benzene on the hematopoietic system was first noted in 1897 by Santesson, and benzene as a bone marrow carcinogen was first recognized by Forni [31]. Exposure to benzene especially high concentrations can damage the hematopoietic function of bone marrow with decreases in the numbers of erythrocytes, leucocytes and thrombocytes in circulating blood, and ultimately result in the onset of aplastic anemia [29]. In the present study, we demonstrated that benzene suppressed the hematopoietic function of bone marrow, and the increase of the expression and activity of Topo IIα treated by TSA or MCP30 contributed to relieve benzene-induced hematotoxicity.

Epigenetics has been reported to be implicated in the regulation of gene expression, proliferation and differentiation of stem cells, and tumorigenesis [32–34]. Because benzene affects the aforementioned processes, it is believed that epigenetic mechanisms play a role in benzene-induced hematotoxicity and tumor formation [35]. Epigenetic modifications of histone typically include acetylation, methylation, phosphorylation, and ubiquitination. The acetylation and methylation of histone play an important role in transcriptional regulation. The abnormal DNA methylation of the promoter region and deacetylation of histone result in the changes of chromosomal structure and the silence of the genes [36]. In this study, we investigated the mechanisms of the relationship of the Topo IIα expression and changes in histone acetylation through ChIP and found that decrease of Topo IIα expression accompanied with decreased acetylation levels of histone H3 and histone H4 in Topo IIα promoter in bone marrow mononuclear cells from benzene-treated mice. The acetylation of histone promotes transcriptional activation of genes, while the deacetylation of histone silences transcription of genes [37]. Therefore, the decreases of acetyl-histone H3 and acetyl-histone H4 of the Topo IIα promoter are one of the mechanisms of decreased expression of Topo IIα induced by benzene.

Dynamic histone acetylation and deacetylation balance occurs via HDAC and histone acetylase (HAT). An increase of HDAC activity is more important than a reduction of HAT activity in vivo [38]. Exposure of benzene induced apoptosis of bone marrow mononuclear cells from rats, and which were reverted by TSA treatment with or without 5-aza (a DNA methyltransferase inhibitor) [39], suggesting that benzene-induced hematotoxicity is associated with epigenetic modifications including the acetylation level of histone. Our previous work has shown that MCP30 inhibits HDAC-1 activity and promotes histone H3 and H4 protein acetylation [19]. Recent studies have shown that intraperitoneal injection of TSA increases the acetylation levels of histone H3 and histone H4 [40]. Our results demonstrated that HQ enhanced the HDAC activity, decreased Topo IIα expression and induced apoptosis in human bone marrow mononuclear cells, and TSA or MCP30 could reverse these HQ-induced effects. *In vivo* treatment with TSA or MCP30 also significantly increased the reduced expression and activity of Topo IIα in bone marrow mononuclear cells from benzene-treated mice, but still less than those of the control mice, confirming that HDAC inhibitors can restore the expression and activity of Topo IIα by increasing the acetylation levels of histone H3 and histone H4 in its promoter.

Additionally, our present study also demonstrated that the mRNA levels of SP1 and C-MYB were reduced, while the mRNA levels of SP3 were elevated in mice with benzene-induced hematotoxicity, suggesting that alterations in other regulatory factors of Topo IIα promoter besides histone acetylation may participate in decreased expression and activity of Topo IIα in mice with benzene-induced hematotoxicity, further confirming our previous results in patients with chronic benzene exposure [12].

In conclusion, our results demonstrate that the expression and activity of Topo IIα are decreased in bone marrow mononuclear cells from benzene poisoning mice, companied by reduced acetylation of histone H4 and histone H3 in Topo IIα promoter, with alterations in mRNA levels of Topo IIα promoter regulatory factors. HDAC inhibitors TSA or MCP30 may reverse aforementioned changes in mice with benzene-induced hematotoxicity and may be a class of promising therapeutic agents to relieve benzene-induced hematotoxicity.

## References

[1] Aksoy M. Hematotoxicity and carcinogenicity of benzene. Environ Health Perspect. 1989; 82: 193–197. 2676498 10.1289/ehp.8982193 PMC1568112

[2] Snyder R, Witz G, Goldstein BD. The toxicology of benzene. Environ Health Perspect. 1993; 100: 293–306. 8354177 10.1289/ehp.93100293 PMC1519582

[3] Linet MS, Yin SN, Gilbert ES, Dores GM, Hayes RB, Vermeulen R, et al A retrospective cohort study of cause-specific mortality and incidence of hematopoietic malignancies in Chinese benzene-exposed workers. Int J Cancer. 2015; 137: 2184–2197. 10.1002/ijc.29591 25944549

[4] Chow PW, Abdul Hamid Z, Chan KM, Inayat-Hussain SH, Rajab NF. Lineage-related cytotoxicity and clonogenic profile of 1,4-benzoquinone-exposed hematopoietic stem and progenitor cells. Toxicol Appl Pharmacol. 2015; 284: 8–15. 10.1016/j.taap.2015.01.016 25645895

[5] Faiola B, Fuller ES, Wong VA, Pluta L, Abernethy DJ, Rose J, et al Exposure of hematopoietic stem cells to benzene or 1,4-benzoquinone induces gender-specific gene expression. Stem Cells. 2004; 22: 750–758. 15342939 10.1634/stemcells.22-5-750

[6] Wang L, He X, Bi Y, Ma Q. Stem cell and benzene-induced malignancy and hematotoxicity. Chem Res Toxicol. 2012; 25: 1303–1315. 10.1021/tx3001169 22540379

[7] Pogorelcnik B, Perdih A, Solmajer T. Recent developments of DNA poisons—human DNA topoisomerase IIalpha inhibitors—as anticancer agents. Curr Pharm Des. 2013; 19: 2474–2488. 23363399 10.2174/1381612811319130016

[8] Baker RK, Kurz EU, Pyatt DW, Irons RD, Kroll DJ. Benzene metabolites antagonize etoposide-stabilized cleavable complexes of DNA topoisomerase IIalpha. Blood. 2001; 98: 830–833. 11468185 10.1182/blood.v98.3.830

[9] Hirabayashi Y, Yoon BI, Li GX, Kanno J, Inoue T. Mechanism of benzene-induced hematotoxicity and leukemogenicity: current review with implication of microarray analyses. Toxicol Pathol. 2004; 32 Suppl 2: 12–16. 15503659 10.1080/01926230490451725

[10] Ji Z, Zhang L, Peng V, Ren X, McHale CM, Smith MT. A comparison of the cytogenetic alterations and global DNA hypomethylation induced by the benzene metabolite, hydroquinone, with those induced by melphalan and etoposide. Leukemia. 2010; 24: 986–991. 10.1038/leu.2010.43 20339439 PMC4353491

[11] Fung J, Hoffmann MJ, Kim DD, Snyder R. Inhibition of topoisomerase II in 32D.3(G) cells by hydroquinone is associated with cell death. J Appl Toxicol. 2004; 24: 183–188. 15211611 10.1002/jat.960

[12] Yu K, Shi YF, Yang KY, Zhuang Y, Zhu RH, Xu X, et al Decreased topoisomerase IIalpha expression and altered histone and regulatory factors of topoisomerase IIalpha promoter in patients with chronic benzene poisoning. Toxicol Lett. 2011; 203: 111–117. 10.1016/j.toxlet.2011.02.020 21382456

[13] Ruthenburg AJ, Li H, Patel DJ, Allis CD. Multivalent engagement of chromatin modifications by linked binding modules. Nat Rev Mol Cell Biol. 2007; 8: 983–994. 18037899 10.1038/nrm2298 PMC4690530

[14] Urnov FD, Wolffe AP. Chromatin remodeling and transcriptional activation: the cast (in order of appearance). Oncogene. 2001; 20: 2991–3006. 11420714 10.1038/sj.onc.1204323

[15] Backs J, Olson EN. Control of cardiac growth by histone acetylation/deacetylation. Circ Res. 2006; 98: 15–24. 16397154 10.1161/01.RES.0000197782.21444.8f

[16] Williams KE, Carver TA, Miranda JJ, Kautiainen A, Vogel JS, Dingley K, et al Attomole detection of in vivo protein targets of benzene in mice: evidence for a highly reactive metabolite. Mol Cell Proteomics. 2002; 1: 885–895. 12488464 10.1074/mcp.m200067-mcp200

[17] Kumar P, Tripathi S, Pandey KN. Histone deacetylase inhibitors modulate the transcriptional regulation of guanylyl cyclase/natriuretic peptide receptor-a gene: interactive roles of modified histones, histone acetyltransferase, p300, AND Sp1. J Biol Chem. 2014; 289: 6991–7002. 10.1074/jbc.M113.511444 24451378 PMC3945360

[18] Mahal K, Kahlen P, Biersack B, Schobert R. 4-(1-Ethyl-4-anisyl-imidazol-5-yl)-N-hydroxycinnamide—A new pleiotropic HDAC inhibitor targeting cancer cell signalling and cytoskeletal organisation. Exp Cell Res. 2015; 336: 263–275. 10.1016/j.yexcr.2015.06.008 26101158

[19] Xiong SD, Yu K, Liu XH, Yin LH, Kirschenbaum A, Yao S, et al Ribosome-inactivating proteins isolated from dietary bitter melon induce apoptosis and inhibit histone deacetylase-1 selectively in premalignant and malignant prostate cancer cells. Int J Cancer. 2009; 125: 774–782. 10.1002/ijc.24325 19384952 PMC3778503

[20] Yoon JH, Kim JK, Rha GB, Oh M, Park SH, Seong RH, et al Sp1 mediates cell proliferation-dependent regulation of rat DNA topoisomerase IIalpha gene promoter. Biochem J. 1999; 344 Pt 2: 367–374. 10567217 PMC1220652

[21] Son MY, Kim TJ, Kweon KI, Park JI, Park C, Lee YC, et al ATF is important to late S phase-dependent regulation of DNA topoisomerase IIalpha gene expression in HeLa cells. Cancer Lett. 2002; 184: 81–88. 12104051 10.1016/s0304-3835(02)00160-x

[22] Hagen G, Muller S, Beato M, Suske G. Sp1-mediated transcriptional activation is repressed by Sp3. EMBO J. 1994; 13: 3843–3851. 8070411 10.1002/j.1460-2075.1994.tb06695.x PMC395297

[23] Brandt TL, Fraser DJ, Leal S, Halandras PM, Kroll AR, Kroll DJ. c-Myb trans-activates the human DNA topoisomerase IIalpha gene promoter. J Biol Chem. 1997; 272: 6278–6284. 9045645 10.1074/jbc.272.10.6278

[24] Hopfner R, Mousli M, Oudet P, Bronner C. Overexpression of ICBP90, a novel CCAAT-binding protein, overcomes cell contact inhibition by forcing topoisomerase II alpha expression. Anticancer Res. 2002; 22: 3165–3170. 12530060

[25] Zhang S, Zhang Y, Zhuang Y, Wang J, Ye J, Zhang S, et al Matrine induces apoptosis in human acute myeloid leukemia cells via the mitochondrial pathway and Akt inactivation. PLoS One. 2012; 7: e46853 10.1371/journal.pone.0046853 23056487 PMC3466205

[26] Han Y, Ye A, Zhang Y, Cai Z, Wang W, Sun L, et al Musashi-2 Silencing Exerts Potent Activity against Acute Myeloid Leukemia and Enhances Chemosensitivity to Daunorubicin. PLoS One. 2015; 10: e0136484 10.1371/journal.pone.0136484 26308531 PMC4550418

[27] Ward CO, Kuna RA, Snyder NK, Alsaker RD, Coate WB, Craig PH. Subchronic inhalation toxicity of benzene in rats and mice. Am J Ind Med. 1985; 7: 457–473. 4003405 10.1002/ajim.4700070510

[28] Yu K, Yang KY, Ren XZ, Chen Y, Liu XH. Amifostine protects bone marrow from benzene-induced hematotoxicity in mice. Int J Toxicol. 2007; 26: 315–323. 17661222 10.1080/10915810701489697

[29] Velasco Lezama R, Barrera Escorcia E, Munoz Torres A, Tapia Aguilar R, Gonzalez Ramirez C, Garcia Lorenzana M, et al A model for the induction of aplastic anemia by subcutaneous administration of benzene in mice. Toxicology. 2001; 162: 179–191. 11369114 10.1016/s0300-483x(01)00371-7

[30] Gieseler F, Bauer E, Nuessler V, Clark M, Valsamas S. Molecular effects of topoisomerase II inhibitors in AML cell lines: correlation of apoptosis with topoisomerase II activity but not with DNA damage. Leukemia. 1999; 13: 1859–1863. 10557063 10.1038/sj.leu.2401570

[31] Forni AM, Cappellini A, Pacifico E, Vigliani EC. Chromosome changes and their evolution in subjects with past exposure to benzene. Arch Environ Health. 1971; 23: 385–391. 5133800 10.1080/00039896.1971.10666024

[32] Esteller M, Herman JG. Cancer as an epigenetic disease: DNA methylation and chromatin alterations in human tumours. J Pathol. 2002; 196: 1–7. 11748635 10.1002/path.1024

[33] Shames DS, Minna JD, Gazdar AF. DNA methylation in health, disease, and cancer. Curr Mol Med. 2007; 7: 85–102. 17311535 10.2174/156652407779940413

[34] Bibikova M, Chudin E, Wu B, Zhou L, Garcia EW, Liu Y, et al Human embryonic stem cells have a unique epigenetic signature. Genome Res. 2006; 16: 1075–1083. 16899657 10.1101/gr.5319906 PMC1557765

[35] Baccarelli A, Bollati V. Epigenetics and environmental chemicals. Curr Opin Pediatr. 2009; 21: 243–251. 19663042 10.1097/mop.0b013e32832925cc PMC3035853

[36] Beerman I, Rossi DJ. Epigenetic regulation of hematopoietic stem cell aging. Exp Cell Res. 2014; 329: 192–199. 10.1016/j.yexcr.2014.09.013 25261778 PMC4250347

[37] Louis M, Rosato RR, Brault L, Osbild S, Battaglia E, Yang XH, et al The histone deacetylase inhibitor sodium butyrate induces breast cancer cell apoptosis through diverse cytotoxic actions including glutathione depletion and oxidative stress. Int J Oncol. 2004; 25: 1701–1711. 15547708

[38] Sonnemann J, Gruhn B, Wittig S, Becker S, Beck JF. Increased activity of histone deacetylases in childhood acute lymphoblastic leukaemia and acute myeloid leukaemia: support for histone deacetylase inhibitors as antileukaemic agents. Br J Haematol. 2012; 158: 664–666. 10.1111/j.1365-2141.2012.09187.x 22686282

[39] Gao A, Zuo X, Song S, Guo W, Tian L. Epigenetic modification involved in benzene-induced apoptosis through regulating apoptosis-related genes expression. Cell Biol Int. 2011; 35: 391–396. 10.1042/CBI20100256 21143203

[40] Avila AM, Burnett BG, Taye AA, Gabanella F, Knight MA, Hartenstein P, et al Trichostatin A increases SMN expression and survival in a mouse model of spinal muscular atrophy. J Clin Invest. 2007; 117: 659–671. 17318264 10.1172/JCI29562 PMC1797603

